# Combined Immunotherapy Improves Outcome for Replication-Repair-Deficient (RRD) High-Grade Glioma Failing Anti–PD-1 Monotherapy: A Report from the International RRD Consortium

**DOI:** 10.1158/2159-8290.CD-23-0559

**Published:** 2023-10-12

**Authors:** Anirban Das, Nicholas R. Fernandez, Adrian Levine, Vanessa Bianchi, Lucie K. Stengs, Jiil Chung, Logine Negm, Jose Rafael Dimayacyac, Yuan Chang, Liana Nobre, Ayse B. Ercan, Santiago Sanchez-Ramirez, Sumedha Sudhaman, Melissa Edwards, Valerie Larouche, David Samuel, An Van Damme, David Gass, David S. Ziegler, Stefan S. Bielack, Carl Koschmann, Shayna Zelcer, Michal Yalon-Oren, Gadi Abede Campino, Tomasz Sarosiek, Kim E. Nichols, Rebecca Loret De Mola, Kevin Bielamowicz, Magnus Sabel, Charlotta A. Frojd, Matthew D. Wood, Jason M. Glover, Yi-Yen Lee, Magimairajan Vanan, Jenny K. Adamski, Sebastien Perreault, Omar Chamdine, Magnus Aasved Hjort, Michal Zapotocky, Fernando Carceller, Erin Wright, Ivana Fedorakova, Alexander Lossos, Ryuma Tanaka, Michael Osborn, Deborah T. Blumenthal, Melyssa Aronson, Ute Bartels, Annie Huang, Vijay Ramaswamy, David Malkin, Adam Shlien, Anita Villani, Peter B. Dirks, Trevor J. Pugh, Gad Getz, Yosef E. Maruvka, Derek S. Tsang, Birgit Ertl-Wagner, Cynthia Hawkins, Eric Bouffet, Daniel A. Morgenstern, Uri Tabori

**Affiliations:** 1 Division of Haematology/Oncology, The Hospital for Sick Children, Toronto, Canada.; 2 Program in Genetics and Genome Biology, The Hospital for Sick Children, Toronto, Canada.; 3 The Arthur and Sonia Labatt Brain Tumour Research Centre, The Hospital for Sick Children, Toronto, Canada.; 4 Department of Paediatric Haematology and Oncology, Tata Medical Center, Kolkata, India.; 5 Department of Paediatrics, University of Toronto, Toronto, Canada.; 6 Department of Paediatric Laboratory Medicine, The Hospital for Sick Children, Toronto, Canada.; 7 Department of Laboratory Medicine and Pathobiology, Faculty of Medicine, University of Toronto, Toronto, Canada.; 8 Institute of Medical Science, Faculty of Medicine, University of Toronto, Toronto, Canada.; 9 Pediatric Haematology/Oncology Department, CHU de Québec-Université Laval, Quebec City, Canada.; 10 Department of Paediatric Oncology, Valley Children's Hospital, Madera, California.; 11 Department of Paediatric Haematology and Oncology, Saint Luc University Hospital, Université Catholique de Louvain, Brussels, Belgium.; 12 Atrium Health/Levine Children's Hospital, Charlotte, North Carolina.; 13 Kids Cancer Centre, Sydney Children's Hospital, Randwick, Australia.; 14 School of Clinical Medicine, UNSW Sydney, Sydney, Australia.; 15 Department of Pediatric Oncology, Hematology and Immunology, Center for Childhood, Adolescent, and Women's Medicine, Stuttgart Cancer Center, Klinikum Stuttgart, Stuttgart, Germany.; 16 Pediatric Hematology/Oncology, C.S. Mott Children's Hospital, University of Michigan, Ann Arbor, Michigan.; 17 Department of Pediatrics, London Health Sciences Centre, London, Canada.; 18 Department of Paediatric Haematology-Oncology, Sheba Medical Centre, Ramat Gan, Israel.; 19 Lux Med Onkologia, Warsaw, Poland.; 20 Department of Oncology, St Jude Children's Research Hospital, Memphis, Tennessee.; 21 Oregon Health and Science University, Portland, Oregon.; 22 Department of Pediatrics, Section of Pediatric Hematology/Oncology, The University of Arkansas for Medical Sciences/Arkansas Children's Hospital, Little Rock, Arkansas.; 23 Department of Paediatrics, Institute of Clinical Sciences, Sahlgrenska Academy, University of Gothenburg & Queen Silvia Children's Hospital, Sahlgrenska University Hospital, Gothenburg, Sweden.; 24 Department of Oncology, Sahlgrenska University Hospital, Gothenburg, Sweden.; 25 Neuropathology, Oregon Health & Science University Department of Pathology, Portland, Oregon.; 26 Department of Pediatric Hematology/Oncology, Randall Children's Hospital, Portland, Oregon.; 27 Department of Neurosurgery, Neurological Institute, Taipei Veterans General Hospital, Taipei, Taiwan.; 28 Pediatric Hematology-Oncology, CancerCare Manitoba, Winnipeg, Canada.; 29 CancerCare Manitoba Research Institute, Pediatrics and Child Health, University of Manitoba, Winnipeg, Canada.; 30 Neuro-oncology Division, Birmingham Children's Hospital, Birmingham, United Kingdom.; 31 Neurosciences Department, Child Neuro­logy Division, CHU Sainte-Justine, Montreal, Canada.; 32 Pediatric Hematology Oncology, King Fahad Specialist Hospital Dammam, Eastern Province, Saudi Arabia.; 33 Department of Paediatric Haematology and Oncology, St. Olav's University Hospital, Trondheim, Norway.; 34 Department of Paediatric Haematology and Oncology, Second Faculty of Medicine, University Hospital Motol, Charles University, Prague, Czech Republic.; 35 Paediatric and Adolescent Neuro-Oncology and Drug Development, The Royal Marsden NHS Foundation Trust & Division of Clinical Studies, The Institute of Cancer Research, London, United Kingdom.; 36 Division of Neuro-Oncology, Akron Children's Hospital, Akron, Ohio.; 37 Clinic of Pediatric Oncology and Hematology, University Children's Hospital, Banská Bystrica, Slovakia.; 38 Department of Oncology, Leslie and Michael Gaffin Centre for Neuro-Oncology, Hadassah-Hebrew University Medical Centre, Jerusalem, Israel.; 39 Division of Hematology/Oncology/Blood and Marrow Transplantation, Department of Pediatrics, Medical College of Wisconsin, Milwaukee, Wisconsin.; 40 Women's and Children's Hospital, North Adelaide, Australia.; 41 Neuro-Oncology Service, Tel-Aviv Medical Center, Sackler Faculty of Medicine, Tel-Aviv University, Tel-Aviv, Israel.; 42 Zane Cohen Centre for Digestive Diseases, Mount Sinai Hospital, Toronto, Canada.; 43 Department of Medical Biophysics, University of Toronto, Toronto, Canada.; 44 Division of Neurosurgery, The Hospital for Sick Children, Toronto, Canada.; 45 Developmental and Stem Cell Biology Program, The Hospital for Sick Children, Toronto, Canada.; 46 Ontario Institute for Cancer Research, Princess Margaret Cancer Centre, Toronto, Canada.; 47 Broad Institute of Harvard and MIT, Cambridge, Massachusetts.; 48 Technion-Israel Institute of Technology, Tel-Aviv, Israel.; 49 Radiation Medicine Program, Princess Margaret Cancer Centre, University Health Network, Toronto, Canada.; 50 Department of Diagnostic Imaging, The Hospital for Sick Children, Toronto, Canada.

## Abstract

Immune checkpoint inhibition (ICI) is effective for replication-repair-deficient, high-grade gliomas (RRD-HGG). The clinical/biological impact of immune-directed approaches after failing ICI monotherapy is unknown. We performed an international study on 75 patients treated with anti–PD-1; 20 are progression free (median follow-up, 3.7 years). After second progression/recurrence (*n* = 55), continuing ICI-based salvage prolonged survival to 11.6 months (*n* = 38; *P* < 0.001), particularly for those with extreme mutation burden (*P* = 0.03). Delayed, sustained responses were observed, associated with changes in mutational spectra and the immune microenvironment. Response to reirradiation was explained by an absence of deleterious postradiation indel signatures (ID8). CTLA4 expression increased over time, and subsequent CTLA4 inhibition resulted in response/stable disease in 75%. RAS–MAPK-pathway inhibition led to the reinvigoration of peripheral immune and radiologic responses. Local (flare) and systemic immune adverse events were frequent (biallelic mismatch-repair deficiency > Lynch syndrome). We provide a mechanistic rationale for the sustained benefit in RRD-HGG from immune-directed/synergistic salvage therapies. Future approaches need to be tailored to patient and tumor biology.

**Significance:**

Hypermutant RRD-HGG are susceptible to checkpoint inhibitors beyond initial progression, leading to improved survival when reirradiation and synergistic immune/targeted agents are added. This is driven by their unique biological and immune properties, which evolve over time. Future research should focus on combinatorial regimens that increase patient survival while limiting immune toxicity.

*This article is featured in Selected Articles from This Issue, p. 201*

## Teaser

Continuous and obligatory mutagenesis in patients with germline DNA replication-repair deficient refractory glioblastoma allows for sustained immune-surveillance and prolonged survival following synergistic immune-directed combinations.

## INTRODUCTION

DNA replication during cell division is an error-prone process (1). The two mechanisms governing fidelity are the mismatch-repair system, which includes PMS2, MLH1, MSH2, and MSH6, and the internal proofreading capability of the DNA polymerases, POLE and POLD1^2,3^. Primary (germline or somatic) defects in either mechanism lead to replication-repair-deficient (RRD; refs. 3, 4) cancers, most frequently high-grade (WHO grades 3 and 4) gliomas (HGG; ref. 5). HGG are the deadliest brain malignancy for which cure is rare despite multimodality management including surgery, radiation, and chemotherapy (6–9). Furthermore, primary RRD-HGG recur rapidly following chemoradiation because of intrinsic resistance, culminating in early fatality (3, 10).

Immune checkpoint inhibition (ICI) blocking the PD1–PDL1 (programmed cell death protein-1 and its ligand) axis has improved outcomes for several advanced cancers over the past decade but have failed in HGG, including those which acquire secondary hypermutation as a result of previous therapy (10). However, the extremely high rates and relatively early accumulation of DNA replication errors in primary RRD-HGG lead to significantly elevated tumor mutation burden (TMB; refs. 4, 11) and microsatellite instability (MSI), which can potentially be targeted using immune-directed therapies (4, 12, 13).

The International Replication-Repair Deficiency Consortium (IRRDC; https://replicationrepair.ca, a collaborative effort of physicians and scientists from >50 countries; refs. 3, 4, 11, 12, 14, 15), has recently reported on the success of ICI in recurrent RRD-HGG (16–18). Outcomes following ICI with single-agent anti–PD-1 were comparable or superior to other adult hypermutated cancers (8–13, 14), with the first-ever objective responses in refractory glioblastoma, translating to a remarkable 3-year overall survival of 39% for tumors failing chemoradiation (16). Nevertheless, the majority of RRD-HGG eventually progress after anti–PD-1 monotherapy (15, 19). Although data on combinatorial immunotherapies are emerging for several adult cancers including advanced melanoma (20, 21), renal cell carcinoma (22), and colorectal carcinomas (23), contributing to improved outcomes compared with ICI monotherapy even as first-line, such data do not exist for RRD-HGG. Furthermore, the mechanisms for any such responses after failing single-agent PD-1 inhibition are not well established.

Here we report the real-world data from the largest registry study of salvage immune-based therapies for RRD-HGG patients, whose tumors had progressed after anti–PD-1 monotherapy. These patients, treated globally across partner institutions of the IRRDC over the past 7 years, provide comprehensive clinical and biological insights into the mechanistic roles and synergism of the different immune-based treatment approaches for these deadly hypermutant cancers.

## RESULTS

### Study Population, Treatment, and Survival

**Figure 1.**
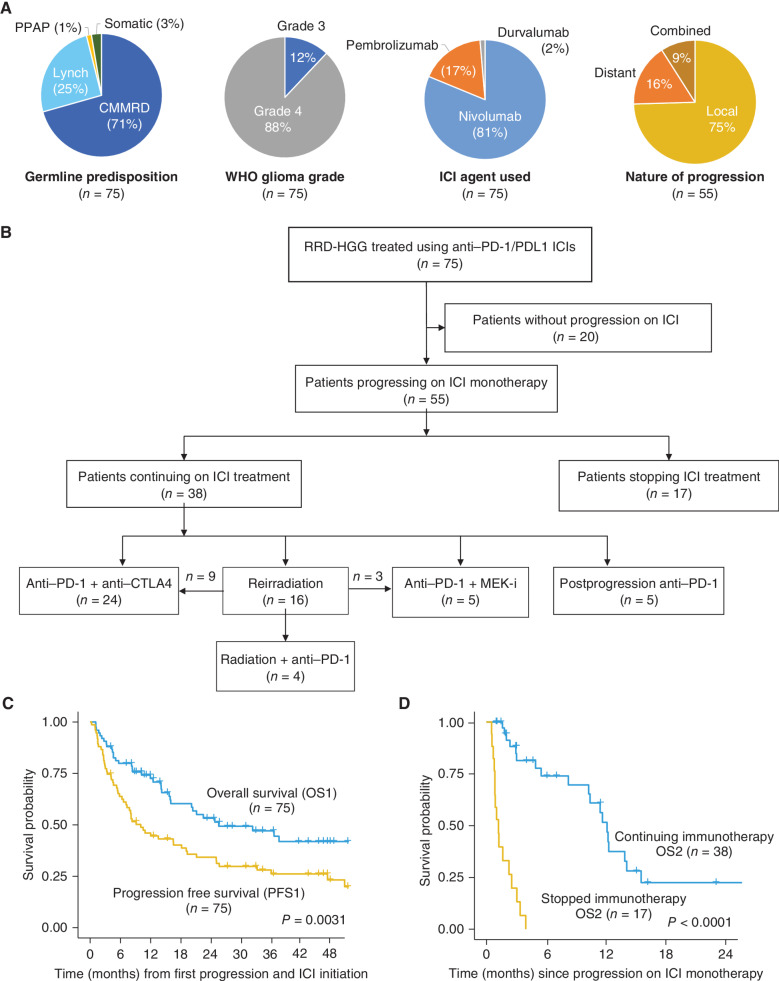
Patients with RRD-HGG treated with ICI (*n* = 75). **A,** Cohort characteristics. **B,** Flow of patients with RRD-HGGs treated with ICIs and salvage regimens. MEK-i, MEK inhibitor. **C,** Progression-free (PFS1) and overall survival (OS1) on anti–PD-1/PDL1 monotherapy. **D,** Overall survival (OS2) for 55 patients progressing on monotherapy stratified by continued (*n* = 38) or no ICI (*n* = 17). PPAP, polymerase proofreading associated polyposis syndrome; CMMRD, constitutional mismatch repair deficiency syndrome.

In this IRRDC study, 75 patients with RRD-HGG were included. They had received treatment with anti–PD-1/PDL1 monotherapy at first progression following the failure of prior chemoirradiation in the majority (*n* = 70; 94%). Glioblastoma (WHO grade 4) was the most common pathologic diagnosis (*n* = 66; 88%). A germline etiology was confirmed in 97% (Fig. 1A; Supplementary Table S1). Nivolumab (81%) and pembrolizumab (17%) were the agents used in the majority of patients (Fig. 1A).

Remarkably, 20 patients (27%) with aggressive, recurrent hypermutant RRD-HGG are still disease free at a median follow-up of 44.6 months [interquartile range (IQR), 29.7–51.1; Fig. 1B]. Unfortunately, 55 (73%) patients’ tumors progressed on ICI monotherapy, most commonly at the local site (75%; Fig. 1A). Among these 55 patients, 17 (31%) discontinued ICI completely, whereas 38 (69%) continued ICI, either alone or with salvage combinations of CTLA4 inhibition, MEK inhibition, and/or reirradiation as per the consortium's guidelines. There was no selection for stopping versus continuing treatment based specifically on the clinical status at the time of progression on monotherapy, and the ultimate decision and choices of agents were with the treating physician (Fig. 1B; Methods; Supplementary Table S2). Specifically, the attributable reasons for stopping anti–PD-1/PDL1 in the 17 patients included the following, namely, the treating physician's decision following radiologic progression alone (*n* = 12/17; 71%), for acute clinical deterioration combined with radiologic progression (*n* = 2/17; 12%), decision by the family to stop after radiologic progression (*n* = 2/17; 12%), and a second malignancy (T-cell lymphoma; *n* = 1). The median follow-up time for the entire cohort (*n* = 75) since start of ICI treatment was 41.1 months (95% CI: 30.6–48) and that for patients with subsequent progression following anti–PD-1/PDL1 monotherapy (*n* = 55) was 15 months (95% CI, 11.3–not reached).

Median overall survival (OS1) for all 75 patients with recurrent RRD-HGG was 25.5 months, more than double the PFS1 of 10 months on anti–PD-1 monotherapy (*P* = 0.005; Fig. 1C). This suggested that salvage therapies could significantly prolong survival among some patients after progression following anti–PD-1 monotherapy. Importantly, for the 55 patients who experienced tumor progression on anti–PD-1 monotherapy, the median survival (OS2) was 11.6 months for those who continued ICI postprogression (*n* = 38), with 18 patients (51%) being alive at the last follow-up. In contrast, median survival for patients discontinuing ICI (*n* = 17) was merely 1.2 months (*P* < 0.001; Fig. 1D) with no survivors, though the majority of the patients were initially clinically stable despite overt radiographic progression. This stark difference in survival suggests the efficacy of continuing immune-directed therapies in these patients.

### Biomarker Analyses

Previously, we had reported that TMB and MSI are independent predictors for response and survival following anti–PD-1 monotherapy in RRD cancers (16). Reports have suggested possible roles for defects in antigen presentation and interferon signaling in determining immune escape following ICI in other cancers (24–27). Notably, we neither observed *B2M* mutations nor identified enrichment for *JAK1/JAK2* mutations in patients who progressed on anti–PD-1 monotherapy (Supplementary Fig. S1A). We also observed that B2M expression was uniformly elevated across RRD-HGG, suggesting that loss of B2M expression is possibly rare in these hypermutant tumors (Supplementary Fig. S1B). Furthermore, loss of heterozygosity (LOH) for HLA-I was rare in RRD-HGG and was not associated with progression on ICI treatment (Supplementary Fig. S1C). Last, we did not observe the enrichment of clonal mutations (28) in those who did not progress (Supplementary Fig. S1D). Instead, we observed that patients who did not progress had a higher peak of the late burst of mutations that we have previously described to be associated with secondary somatic polymerase-proofreading deficiency (PPD) in MMR-deficient cancers (11) and has an association with improved responses to ICI treatment (Supplementary Fig. S1E; refs. 16, 29). These initial observations need to be systematically analyzed in larger cohorts.

**Figure 2.**
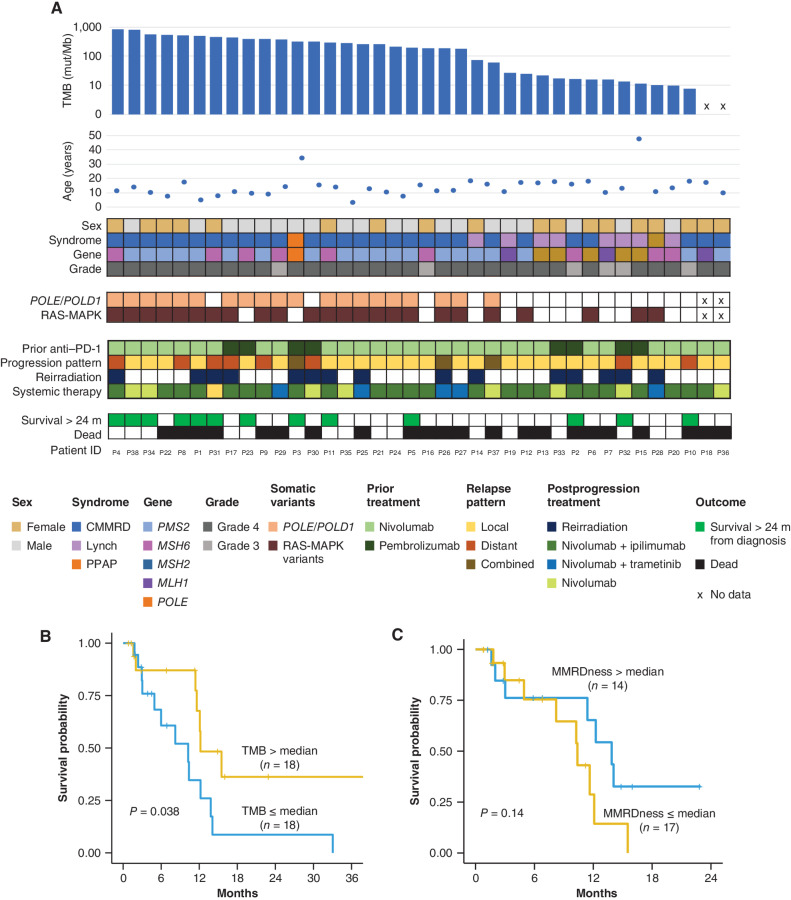
Genomic features of patients with RRD-HGG treated with salvage therapies (*n* = 38). **A,** Onco-plot summarizing clinical and genomic features of patients who progressed on anti–PD-1 monotherapy and continued ICI treatment. **B,** Impact of TMB on survival. **C,** Impact of genomic MSI as measured by the tumor MMRDness score on survival (Methods).

Detailed clinical, genomic, and molecular analyses were additionally performed on the patient cohort continuing salvage treatments with ICI after failing anti–PD-1 monotherapy (Fig. 2A; Supplementary Table S2). This confirmed the extreme mutation burden in all patients (median TMB: 199.65 mutations/Mb; IQR, 20.4–386.1). Patients with TMB below median were more likely to have heterozygous Lynch syndrome as opposed to homozygous constitutional mismatch-repair deficiency (CMMRD; 44% vs. none; *P* = 0.002). Similarly, RRD-HGG characterized by TMB below median were less likely to harbor *POLE, POLD1* somatic mutations (ref. 4; 22% vs. 89%; *P* = 0.0001), or RAS–MAP kinase somatic aberrations (ref. 15; 44% vs. 89%; *P* = 0.01; Fig. 2A). Remarkably, a higher TMB (>median) remained an important determinant of improved overall survival for salvage therapies even within this cohort of hyper and ultra-hypermutant cancers (*P* = 0.03; Fig. 2B). However, unlike at first progression following monotherapy (16), genomic MSI, measured by the tumor MMRDness scores (Methods; ref. 30) failed to significantly stratify post-salvage survival once patients had progressed after anti–PD-1 monotherapy (Fig. 2C). In patients who had a second specimen available for sequencing after failing ICI treatment, we did not find enrichment for *B2M, JAK1*, or *JAK2* mutations at progression (Supplementary Fig. S2A). Neither did we observe the emergence of LOH in HLA-I. Although we observed a definite evolution in the mutational spectrum (Supplementary Fig. S2B), we did not witness a consistent pattern of change in mutational clonality, suggesting that mutation accumulation in RRD-HGG may be stochastic, and clonal evolution does not necessarily herald immune evasion (Supplementary Fig. S2C). These data suggest that for RRD-HGG with extreme TMB and MSI, distinct mechanisms of immune evasion may be operational (24, 31, 32) and need to be explored in future studies.

### Addition of a CTLA Inhibitor Improves Survival and Reveals Critical Biological Mechanisms Responsible for Response and Toxicity Following Dual ICI in Germline RRD

**Figure 3.**
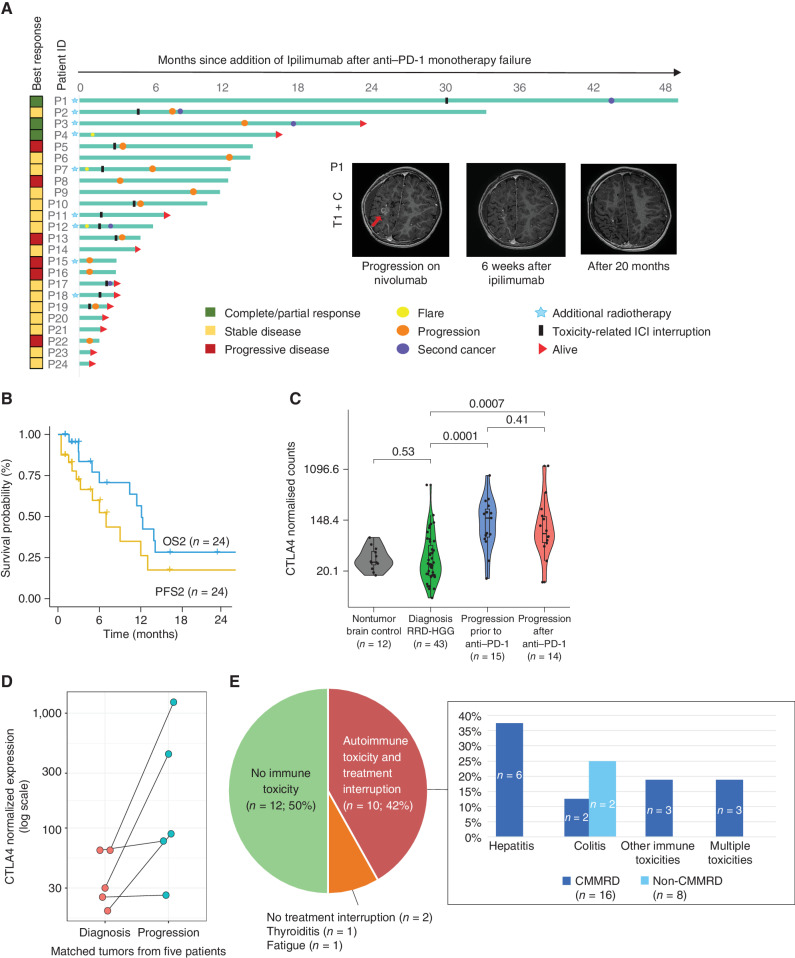
Patients receiving dual-checkpoint inhibition with ipilimumab and anti–PD-1 after failing ICI monotherapy (*n* = 24). **A,** Swimmer's plot for each patient, showing the best documented radiologic response at any time during treatment. Inset, representative radiologic image showing response to dual-checkpoint inhibition. **B,** Progression-free (PFS2) and overall survival (OS2). **C,** Normalized CTLA4 expression counts generated using NanoString platform (Methods) for in-house non-RRD, nonmalignant brain controls, and at different time points for RRD-HGG. **D,** Paired analysis of normalized CTLA4 expression counts before and after anti–PD-1 therapy for the same patient. **E,** Toxicities (≥CTCAE grade 3) observed in CMMRD and Lynch syndrome patients on dual ICI.

Ipilimumab was added to anti–PD-1 in 24 (63%) patients. Radiologically, disease control was noted in 18 out of 24 (75%) tumors, with three (12.5%) patients demonstrating complete or partial responses (Fig. 3A), and 15 (62.5%) achieving disease stability. This translated to second overall survival (OS2) on dual ICI of 12.1 months (Fig. 3B), with postprogression survival >20 months observed in three patients (Fig. 3A; Supplementary Table S2).

Analysis of immune biomarkers using a NanoString-3D panel (Methods) revealed a significant increase in CTLA4 (33) expression, both at progression following chemoradiation (*P* = 0.001) and after failing anti–PD-1 monotherapy (*P* = 0.007; Fig. 3C). Intriguingly, analysis of paired samples before and after single-agent PD-1 inhibition showed an increase in CTLA4 expression (Fig. 3D). Together, these observations suggested ongoing infiltration of lymphocytes during therapy through disruption of the blood–brain barrier and subsequent compensatory upregulation of the coinhibitory pathways like CTLA4 following PD1 inhibition. A similar phenomenon has been previously observed in select non-RRD cancers (34, 35).

Toxicity was a major challenge (50%), with significant immune-related adverse effects (irAE) leading to interruption of ICI, followed by immunosuppressive therapies in 10 (42%) patients (Fig. 3E; Supplementary Table S2). Hepatitis (60%) and colitis (33%) were the most frequent irAEs responsible for treatment interruption. The overall incidence of irAE was higher than previously reported using dual ICI treatment at the recommended dose regimen (36, 37). Hence, we investigated whether the incidence could be affected by the germline defect in our patients and whether there would be differences between patients with CMMRD (*n* = 16) versus heterozygous Lynch syndrome (*n* = 8). Colitis, the most frequent serious irAE previously reported with anti-CTLA4 therapy (38), was seen in both syndromes. However, all other irAEs, specifically the high incidence of hepatitis (37.5%) and multiple irAE (18.7%), were restricted to patients with CMMRD. Despite limited numbers, these data can plausibly be explained by the ongoing mutagenesis and increased MS indels observed even in normal cells of CMMRD patients harboring complete loss of MMR function (29), resulting in higher immunogenicity and incidence of irAEs in other organs in these patients.

### Combined Immune and Targeted Therapy Exhibit Evidence of Immune Synergism in Patients with RRD-HGG

**Figure 4.**
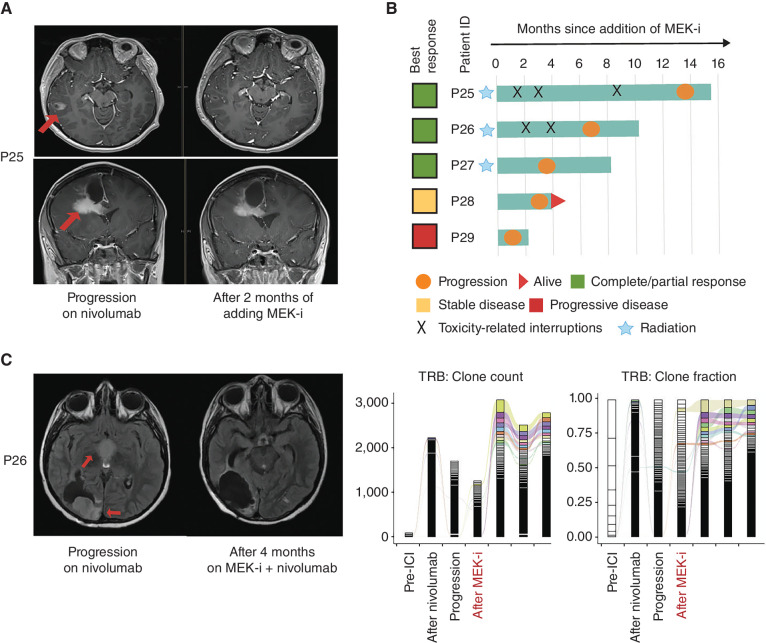
Patients treated with nivolumab and trametinib (*n* = 5). **A,** Objective responses in bifocal glioblastoma progressing on nivolumab. **B**, Swimmer's plot summarizing events in each patient. **C**, The patient progressing at primary and distant sites on nivolumab had complete response on being simultaneously treated with radiation and addition of trametinib to nivolumab. T-cell receptor (TCR) clonotype analysis shows an initial increase in TCRB after starting on nivolumab, and invigoration of response at salvage treatment postprogression. This was observed both in terms of increased absolute clonal counts after adding trametinib, as well as clonal selection, plausibly for more tumor-specific TCR clones (as shown by the colored bars), as this correlated with the response seen in radiology. MEK-i, MEK inhibitor.

Our previous data demonstrated that *RAS/MAPK* mutations are enriched in RRD-HGG, and their allelic frequency increases over time, leading to transcriptional activation of the pathway and susceptibility to targeted inhibition in preclinical experiments (15). Because MEK inhibition also resulted in immune activation in select patients with non-RRD cancers, (39–41), we hypothesized that MEK inhibitors could be effective in RRD-HGG harboring *RAS/MAPK* variants and, in addition, contribute to immune synergism to drive the responses in patients with RRD-HGG continuing on anti–PD-1. Hence, in our current study, we analyzed five patients treated with the addition of MEK inhibitor (trametinib) upon failure of anti–PD-1 monotherapy. The tumors in all five patients harbored multiple *RAS–MAPK* variants, including pathogenic, frameshift variants in *NF1* with allele frequency at diagnosing exceeding 0.4. Remarkably, the addition of trametinib to PD1 inhibition revealed objective responses in 3 out of 5 (60%) patients (Fig. 4A–C), achieving a median OS2 of 10 months (Fig. 4B; Supplementary Table S2). Specifically, we observed robust reinvigoration of the peripheral immune response surpassing the immune activation that was seen after the initiation of anti–PD-1 monotherapy (Fig. 4C). Using T-cell receptor clonotype analysis (16), an increase in the T-cell repertoire was observed, including higher clone count and diversity, with selected original clones expanding on combinatorial treatment, suggesting a more robust and specific immune response to the tumor after adding trametinib. Strikingly, this corresponded with objective radiologic response in the patient (P26; Fig. 4C). Unfortunately, this patient also had significant autoimmune toxicity after the addition of trametinib, including myositis that was refractory to nonsteroidal anti-inflammatory treatment. He had previously tolerated nivolumab monotherapy without autoimmune side effects. These pilot observations on the immune synergism of MEK and checkpoint inhibition need to be validated in larger cohorts of RRD-HGG patients.

### Clinical and Biological Impact of Reirradiation and Immunotherapy in Recurrent RRD-HGG

**Figure 5.**
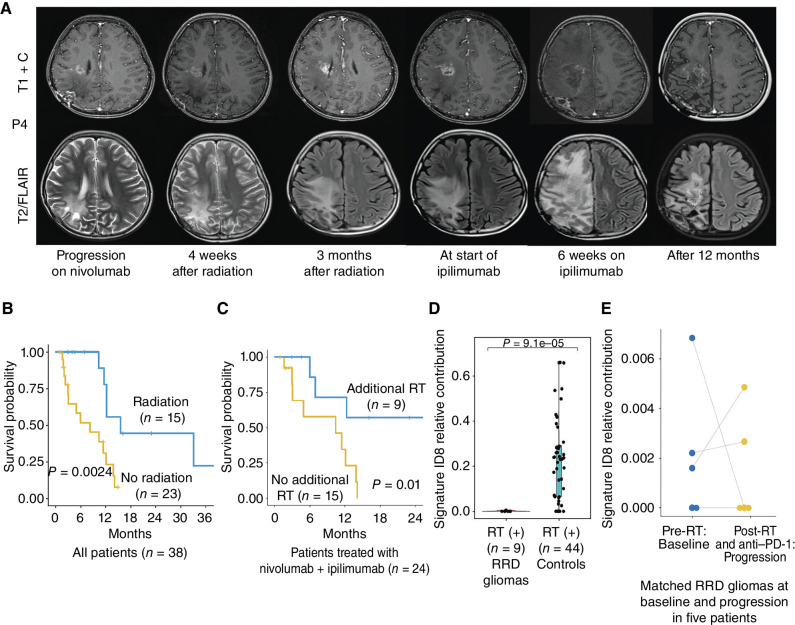
Impact of radiotherapy. **A,** Patient progressing on nivolumab received reirradiation, following which further progression prompted the addition of ipilimumab. This was followed by radiologic flare, and continued treatment with supportive care led to delayed response. **B,** Impact of reirradiation on survival. **C,** Impact of additional reirradiation (RT) in patients who received nivolumab and ipilimumab. **D,** Relative contribution of the radiation-induced indel signature (ID8) in RRD-HGG and controls from the GLASS cohort (Methods). **E,** Paired analysis of the relative contribution of ID8 before (at diagnosis) and after the second progression (post primary radiation, and then immunotherapy at first progression) in five patients.

Among the 38 patients continuing ICI after progression, 15 (39%) received reirradiation. These included patients treated with combinatorial therapies with anti-CTLA4 and MEK inhibitor. We noted that 3 out of 9 (33%) tumors treated with received reirradiation demonstrated radiologic “flare” (16) at a median time of 17 days after starting ipilimumab (Fig. 5A). As this was not observed in patients receiving the combination without reirradiation, it is possible that reirradiation could upregulate the immune response to dual ICI. Delayed responses and prolonged OS2 (median 12 months) were noted in all three patients. Overall, patients receiving reirradiation had a superior median OS2 of 15.5 months as compared with 8.2 months for those who did not (*P* = 0.002; Fig. 5B). Among patients receiving dual ICI, those who received additional reirradiation had an improved OS2 of 33 months, as compared with 10 months for the 15 patients who did not receive reirradiation (*P* = 0.01; Fig. 5C). A similar impact of reirradiation was observed for combined anti–PD-1 and trametinib, which likely did not reach statistical significance because of small numbers (*P* = 0.06; Supplementary Fig. S3A). Interestingly, reirradiation was specifically associated with improved survival for patients with gliomas harboring lower TMB, which are usually less responsive to ICI monotherapy (*P* = 0.05; Supplementary Fig. S3B).

We hypothesized that the improved responses following reirradiation could be related to specific changes in the mutational spectra of RRD-HGG. It has been previously reported that ionizing radiation treatment of glioblastoma leads to double-stranded breaks that are suboptimally repaired via the canonical nonhomologous end joining (C-NHEJ) pathway, resulting in specific small deletions, identified as ID8 (COSMIC; ref. 42). Although indels can be immunogenic because of their frameshift effect (43), the specific acquisition of the ID8 signature was also shown to result in the loss of sensitivity to further radiotherapy (42). We sought to investigate the potential role of irradiation in response to therapy by analyzing the ID8 signature in RRD-HGG that had recurred after treatment with radiation. We observed the absence of ID8 signature in our post-radiation RRD-HGG tumor specimens after treatment with anti–PD-1 monotherapy as compared with previously published post-radiation non-RRD glioma controls not treated with ICI (ref. 42; Fig. 5D). Furthermore, comparison of paired specimens of RRD-HGG did not reveal changes in ID8 mutagenesis (Fig. 5E), pre- and post-ICI, in these patients. This demonstrated that RRD-HGG, which progressed following radiation and subsequent anti–PD-1 treatment, do not harbor signatures of prior radiation exposure. This suggested either retained sensitivity of these emergent clones to ionizing radiation, following the possible elimination of clones harboring ID8 because of, their higher immunogenicity, and ongoing immune editing (44). These observations need to be corroborated in larger studies.

### Continuation of Anti–PD-1 as Monotherapy after Progression Reveals Insights into Immune Evolution in RRD-HGG

**Figure 6.**
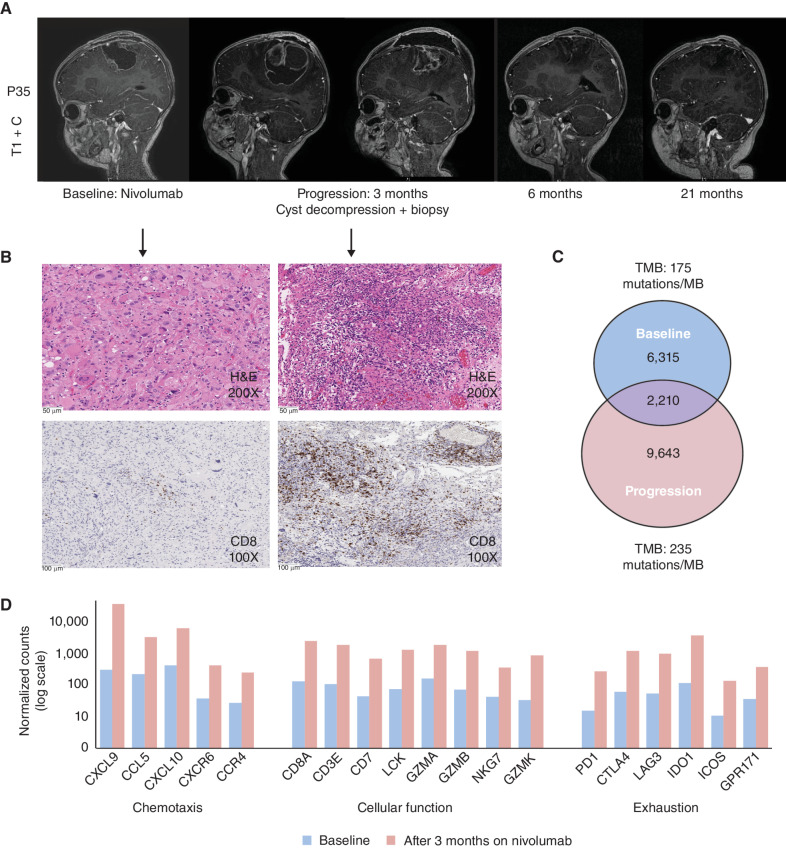
Delayed response after initial progression on continued anti–PD-1 monotherapy. **A,** Delayed response after initial progression on nivolumab monotherapy. **B,** Cyst decompression and biopsy at progression showed a more mitotically active and higher grade tumor with higher TMB and increased CD8 T-cell infiltration. **C,** Mutation overlap showed minimal overlap of the somatic mutational spectrum between the two time points (200X magnification; scale bars represent 50 µm). **D,** Normalized expression counts of immune markers before initiation and after progression on anti–PD-1 monotherapy using the NanoString platform (100X magnification, scale bars represent 100 µm; Methods).

Five patients continued single-agent ICI therapy despite tumor progression with individual modifications (Supplementary Table S2), including switching agents, interruptions due to toxicity, or additional surgical interventions. Of note, 3 out of 5 (60%) patients are alive and in remission while continuing only on anti–PD-1 monotherapy despite showing true initial progression over several months. All three patients had CMMRD and harbored secondary somatic DNA polymerase-proofreading mutations (4), resulting in ultra-hypermutant glioblastoma (P34, P35, and P38 with TMB 559, 275, and 800 mutations/Mb, respectively). Patient P35 had started on nivolumab at glioblastoma progression. This tumor continued to progress over 3 months of ICI with worsening symptoms, prompting a surgical intervention (Fig. 6A). Continuous PD1 blockade post subtotal resection resulted in complete remission of 18 months at the time of writing of this report. P34 experienced continuous multifocal progression of his glioblastoma 4 months after initiation of PD-1 inhibition, resulting in treatment discontinuation. Improved symptoms over the following 6 months prompted an MRI, which remarkably demonstrated an objective response and minimal evidence of disease. ICI was restarted and continued for an additional 18 months, and the patient is alive in complete remission more than 6 months after stopping therapy (>30 months postprogression). The third patient (P38) had rapid progression within 3 months after stopping ICI due to autoimmune side effects. Following subtotal resection and reinitiation of PD-1 blockade, the patient remains clinically well with stable disease, 6 months from the second progression.

We hypothesized that such delayed sustained responses could be related to obligatory ongoing clonal evolution of RRD gliomas, thereby affecting the immune microenvironment. Hence, we analyzed matched samples from the same patient who had tumor resection upon progression on anti–PD-1 monotherapy (P35). Indeed, paired analysis of specimens before and after PD1 inhibition revealed a more aggressive morphology with not only higher mitotic activity but also a higher CD8 T-cell infiltration (Fig. 6B). This was accompanied by higher TMB and minimal mutational overlap in comparison with the baseline sample (Fig. 6C). Analyses of the immune microenvironment using the NanoString immune-panel platform (Methods) demonstrated a several-fold increase in expression of chemotaxis, effector cell, and immune checkpoints including PD1 at progression (Fig. 6D).

These remarkable observations prompted us to investigate the evolution of the inflammatory milieu in RRD-HGG over time in a select cohort of patients who had additional tissue available at progression. Indeed, our pilot data suggest that RRD-HGG can acquire a more proimmune TME over time (Supplementary Fig. S4A and S4B), as their mutational spectra simultaneously evolve (Supplementary Fig. S4C). Furthermore, in one patient (P07) where T-cell receptor (TCR) clonotype analysis was performed on anti–PD-1 monotherapy (Supplementary Fig. S5A), we observed enrichment in clone count (Supplementary Fig. S5B) and specific clonotypes (Supplementary Fig. S5C) even before the initiation of additional salvage therapy. A subsequent biopsy performed days later confirmed novel mutation accumulation (Supplementary Fig. S5D) and an increase in CD8-positive T cells in the microenvironment (Supplementary Fig. S5E). Not surprisingly, the patient subsequently demonstrated an objective radiologic response (Supplementary Fig. S5A).

Overall, these observations (Fig. 6; Supplementary Figs. S2–S5) demonstrated that the changing mutational spectra in RRD-HGG affect the tumor, its microenvironment, and the peripheral immune response, providing a mechanistic rationale for the continued, albeit delayed response to continued anti–PD-1 monotherapy. These initial observations need to be studied systematically in larger numbers of patients.

## DISCUSSION

This registry study from the International RRD Consortium demonstrates both objective responses and improved survival for children and young adults with RRD-HGG, who continued salvage immune-based combinations upon progression on anti–PD-1/PDL1 monotherapy. Further, several important biological and clinical observations became apparent for these RRD-driven, hypermutant cancers, providing insights with possible implications beyond this cohort of patients with a historically rapidly fatal disease (13).

Perhaps the most intriguing observation from our study is the sustained impact of continued immune-based therapies in the context of evident tumor progression and initial resistance. RRD cancers lack the ability to repair mistakes during replication resulting in ongoing mutation accumulation affecting clonal evolution (refs. 4, 11, 15; Fig. 6C; Supplementary Figs. S4C and S5D). This obligatory generation of novel antigenic epitopes and the continuous genomic instability can generate immune surveillance and responses in initially resistant tumors, which is different from other brain cancers that fail ICI. In RRD cancers with extreme TMB and MSI, some mechanisms of immune resistance previously described in lung (45) and other cancers, including defects in antigen presentation (24), interferon signaling (26), and lack of clonality (28), may not be important drivers of immune evasion (refs. 24, 31, 32; Supplementary Figs. S1 and S2). Delayed responses were therefore not unusual in patients with genomically unstable RRD-HGG accumulating extreme TMB and MSI, and these included those with bulky (Fig. 6; Supplementary Fig. S5) as well as multifocal disease (46). Furthermore, as standard radiologic assessment of disease burden and immune responses can be challenging in these situations (16, 46, 47), novel techniques need to be explored to study these dynamic changes to allow robust impact on clinical management (48).

Second, to our knowledge, this is the first comprehensive report on immune-based combinations in brain cancers driven by RRD. Specifically, the role of adding CTLA inhibition to RRD-HGG that fail PD-1 blockade is still relatively unknown. Interestingly, we witnessed both responses and/or disease stability after adding ipilimumab in patients both progressing early and >12–24 months following monotherapy, with prolonged ongoing survival (Fig. 3; Supplementary Table S2). The elevated expression of the inhibitory immune-checkpoint CTLA4 in RRD-HGG post PD-1 blockade provides a strong biological rationale for these secondary responses.

Third, RRD hypermutant cancers have been recently reported to be enriched for *RAS/MAPK* mutations, with targeted inhibition conferring benefit both in vitro and in vivo (15). Responses were seen in patients in our cohort following addition of MEK inhibitors to anti–PD-1 therapy, along with the evidence for reinvigoration of peripheral immune response, suggesting plausible synergism with ICI. MEK inhibition in melanomas and NSCLC leads to increased expression of immunogenic antigens, upregulation of immune-critical molecules, including HLA class I, and increased immune responses in the form of CD8+ T cells in the microenvironment (39–41). Hence, this combination approach will now be explored in a prospective RRD-HGG trial.

Next, we observed an additive effect of reirradiation on survival in patients receiving salvage immune-directed therapies, especially for hypermutant gliomas with TMB < 200 mutations/Mb (Fig. 5; Supplementary Fig. S3). Radiation is reported to synergize and even lead to abscopal effects in conjunction with immunotherapy in adult cancers and animal models, including in glioblastoma (49–53). This synergism plausibly contributed to early radiologic tumor flare (54) in one-third of patients who received reirradiation and dual-checkpoint inhibitors, within days to weeks of starting ipilimumab. Importantly, none of these patients had previously shown pseudoprogression on monotherapy. Remarkably, radiation-specific indel signatures (ID8) were not detected in recurrent RRD-HGG progressing on anti–PD-1 after prior treatment with definitive radiotherapy, suggesting eradication of these clones either due to sensitivity to reirradiation or immune editing of ID8-related mutations. In contrast, ongoing mutagenesis (4) possibly contributed to added immunogenicity that could explain the delayed responses observed in these patients.

Last, we observed that immune-related toxicity was higher than in previously reported studies in both adults and children (36, 37, 55, 56). It was intriguing to note that the high incidence of irAEs in patients treated with dual ICI was enriched in patients with CMMRD as compared with Lynch syndrome, suggesting that hypermutagenesis in normal tissues seen in such patients (29, 30) could contribute to off-target immune effects. This enhanced autoimmunity observed upon reirradiation and MEK inhibition further supports immune activation following these modalities when combined with ICI.

In summary, our study highlights the importance of continuous immune surveillance in RRD cancers, which enables delayed and secondary response after the initial failure of immunotherapy. This is distinct from the irreversible ongoing resistance to chemotherapy and targeted and radiation therapies that is observed in most genomically stable cancers. Specifically, we found that previously described mechanisms of immune evasion may not play a major role in the context of replication-repair deficiency. In this context, the demonstration of the impact of continued immune-directed therapies for this subset of HGG is important, as RRD is increasingly diagnosed as an important driver mechanism in childhood, adolescent, and young adult gliomas (57) and as conventional treatment using drugs like temozolomide are ineffective for these tumors (3, 58, 59). This study was not part of a prospective clinical trial and included relatively low numbers of these rare patients, potentially resulting in heterogeneity and bias. In addition, emerging determinants of response to ICI treatment, including tumor burden, host immune health, and microbiome status, were not formally evaluated. Despite these limitations, observations of the synergism of reirradiation and the beneficial impact of combination of two ICI agents and/or targeted agents in this pilot cohort of patients add to our biological insights and provide a rationale for exploring these approaches in prospective trials. Finally, our observations regarding the unique spectra of immune toxicity depending on the underlying germline predisposition suggest the need for future combinatorial clinical trials to be tailored to both patient and tumor biology. Trials based on these data are currently in various stages of development by international consortium groups.

## METHODS

### Study Design

This global registry study was conducted by the International Replication-Repair Deficiency Consortium (IRRDC; https://replicationrepair.ca) centered at The Hospital for Sick Children (SickKids) in Toronto. Patients confirmed to have a diagnosis of DNA replication-repair deficiency (RRD), a high-grade glioma (HGG; WHO grades 3 and 4), and treated using ICI therapy between 2015 and 2021 were included. Management plans for individual patients were finalized following discussions between individual patient teams and the IRRDC multidisciplinary tumor board at SickKids Toronto, as detailed below. Monthly and ad hoc meetings were coordinated to track progress, address safety concerns as per published guidelines and/or ongoing clinical trial protocols, and collect data in real time. Samples for companion biomarker studies were collected prospectively before and on therapy following written informed consent at specified time points. The study was approved by the SickKids Institutional Research Ethics Review Board (number: 1000048813) and performed following the guidelines provided in the Declaration of Helsinki. Written informed consent was obtained from patients and families, including for the submission of clinical and imaging data, and tissue and blood samples for centralized analysis by the treating physicians. The data cutoff for analysis was February 2022.

### Patients

Patients who were confirmed to have RRD and a diagnosis of HGG (WHO grades 3 and 4) were eligible. The diagnosis of RRD, including the germline diagnosis of CMMRD, Lynch syndrome, or PPD syndrome, was confirmed by the IRRDC's genetic counselor, based on the family history, next-generation panel sequencing of germline samples for MMR and *POLE/POLD1* genes (performed locally or centrally at CLIA-approved laboratories), and immunohistochemical (IHC) staining pattern of the tumor and noncancerous cells. The diagnosis of HGG was confirmed using a central pathology review.

### Treatment Plan

Initial ICI treatment for patients with RRD-HGG involved treatment using anti–PD-1, either nivolumab 3 mg/kg q2-weeks (maximum = 240 mg/dose) or pembrolizumab 2 mg/kg q3-week (maximum = 200 mg/dose). The choice of ICI agent was dependent on local logistics and physician choice. Because of country-specific drug availability issues, a single patient received treatment using durvalumab 10 mg/kg q2 weeks.

Upon progression, reirradiation was advocated where feasible depending on the field, dose, and duration from previous radiotherapy, using published guidelines (60), and under guidance from IRRDC's radiation oncologist. Briefly, patients who were at least 6 months from their initial radiation were considered for reirradiation, with fields ranging from focal radiation (30–54 Gy) for localized disease to whole brain irradiation (30.6 Gy) for those with multifocal disease, in conventional 1.8 Gy daily fractions.

Subsequently, depending on molecular characteristics and biomarkers in the tumor and drug availability, the IRRDC tumor board recommended the addition to anti–PD-1 immunotherapy of either CTLA4 (cytotoxic T-lymphocyte-associated protein-4) inhibition using ipilimumab 1 mg/kg q3 weeks for 4 doses or targeted inhibition of the RAS–MAPK pathway using the MEK-inhibitor trametinib (0.025 mg/kg daily; maximum = 2 mg/dose). Treatment with anti–PD-1 (nivolumab 3 mg/kg q2 weeks, maximum = 240 mg/dose, or pembrolizumab 2 mg/kg q3 weeks, maximum = 200 mg/dose) was continued with these salvage treatments. Although the choice between these salvage options was left to the treating physician team based on logistics and drug availability, the MEK inhibitor was offered only to those harboring alterations in the RAS–MAPK pathway. Though patients were not formally treated as part of a clinical trial, for those continuing on treatment, monthly and as-needed meetings were coordinated to monitor the clinical course prospectively, review radiology, address safety concerns, and collect data in real time. Modifications to the above regimens, if needed, were centrally reviewed by the IRRDC tumor board and were performed as per guidelines provided by the IRDDC, based on specific ongoing clinical trial protocols (NCT02992964, NCT04500548, and NCT03363217).

### Response Assessment

Response and progression were evaluated centrally using the Immunotherapy Response Assessment in Neuro-Oncology (iRANO) criteria (47). Clinically significant toxicities (Common Terminology Criteria for Adverse Events; CTCAE v5.0 ≥grade 3) and treatment interruptions reported by treating physicians were centrally reviewed. Clinical updates regularly shared by treating physicians were also reviewed, and real-time data on treatment initiation, responses, toxicities, and survival outcomes (including date of disease progression and/or patient death) were recorded for all patients.

### Whole-Exome Sequencing and Variant, Signature Calling, and Clonality

Matched tumor and blood whole-exome sequencing (WES) was performed at the Centre for Applied Genomics (TCAG) at SickKids, with variant calling and identification being performed using standard methods as previously reported (16). Briefly, genomic DNA, along with matched germline blood samples for available cases, was extracted using the PaxGene Blood DNA Extraction Kit (cat No./ID: 761133) for blood samples, Qiagen DNeasy Blood & Tissue Kits (cat No./ID: 69504) for frozen tissue, MasterPure Complete DNA, and RNA Purification Kit (Epicentre #MC85200) for paraffin-embedded tissue. WES was performed at TCAG, SickKids, using SureSelect Agilent All Exon v5 kit, followed by sequencing (150×) on Illumina HiSeq 2500. The software bcl2fastq2 v2.17 was used to generate raw fastq files. Alignment to the hg38 reference genome, followed by preprocessing and QC, was adapted from the GATK standard pipeline, using BWA-MEM 0.7.12 (alignment), BAMQC, and Picard 2.6.0 (QC). Somatic variant calling was done post-alignment, using processed bam files from tumor and matched normal samples to call both single-nucleotide variants (SNV) and insertion–deletion (indel) variants. A consensus vcf file of shared variants across 2 or more of 4 variant callers (Mutect v1.1.5, GATK v3.6/Mutect2, Strelka v1.0.14, and Varscan2 Somatic v2.4.2) was generated for SNVs and indels separately, using VCFtools 0.1.15, and these vcfs were annotated using VEP v83. The TMB (SNVs per megabase) from WES data was calculated by counting the total number of somatic SNVs divided by the total number of callable bases in megabases (∼50 Mb). In patients where WES could not be performed due to limited tissue, panel sequencing data from clinically approved platforms were used to estimate TMB and identify variants of interest. The latter included driver secondary somatic mutations in *POLE* and *POLD1* (11) and pathogenic/likely pathogenic somatic variants in the RAS–MAP kinase pathway (15), including *NF1*, *PTPN11*, *RAF, NRAS*, *KRAS*, and *BRAF*. SigProfiler was used to extract mutational signatures (61). Absolute and relative contributions of mutational signatures were determined using modified functions from the MutationalPatterns v.3.8.1 R package, as previously described (42), by fitting to single-base substitutions and indel (ID) context sets from COSMIC v.3.2. Genes related to antigen presentation and interferon signaling for ICI effect and previously described as markers of immune resistance/escape were analyzed (*B2M, JAK1, JAK2, IFNGR1, IFNGR2, TYK2, STAT1, STAT2, STAT5A, STAT5B,* and *IRF*; refs. 26, 27). We used previously published methods (62) to determine the clonal status of each mutation. Specifically, samples with WES for paired tumor and germline were analyzed with ASCAT (v.3.1.2) and alleleCounter (v.3.3.1) to determine tumor purity and ploidy using default parameters with gamma = 1 (62). Cancer cell fraction (CCF) was calculated as defined as (63):




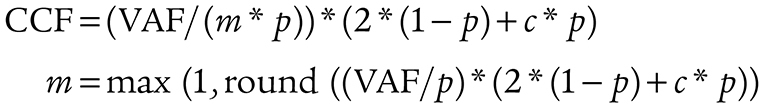




Here *m* is the multiplicity of the mutation, *c* is the local copy number, and *p* is the tumor purity. SNVs and indels with a CCF higher than 0.85 were defined as clonal mutations. The controls for the indel signature analyses were requested from the previously published Glioma Longitudinal Analysis (GLASS) cohort (42).

### HLA Typing

HLA genotyping for paired tumor and normal WES pairs was completed using OptiType 1.3.5 with default parameters (64). We determined the status of somatic HLA-A, HLA-B, and HLA-C using LOHHLA with default parameters but adapted for use with GRCh38 genome assembly by altering the genomic coordinates and alternate alleles in LOHHLAscript.R (45). Samples with a paired unique *P* value less than 0.01 alone were defined to have LOH for that HLA allele. To avoid under-calling of LOH given the extreme paucity of copy-number losses in hypermutation-driven RRD-HGG, a combined cutoff including a copy number of <0.5 and *P* < 0.01 was not used. Cases with errors were excluded.

### Low-Pass Genomic Instability Characterization

Low-pass whole-genome sequencing was performed on tumor tissues to enable the recently published functional low-pass genomic instability characterization assay (29, 30). Briefly, MMRDness scores were calculated using the proportion of number reads with a single base deletion in “A” microsatellites. The log_10_ of the sum of mutated microsatellite proportions in loci 10–15 bases long was calculated, and a scalar value of 1.1 was added.

### Tumor Immune Microenvironment Analysis

Gene-expression profiling was carried out using the NanoString nCounter system. A major advantage of the NanoString platform (compared with RNA sequencing, for example) is its excellent results on formalin-fixed paraffin-embedded tissue (FFPE) as samples were transported from multiple centers from around the world. Total RNA was extracted from FFPE as per the manufacturer's instructions (CellData). RNA concentration was measured (Nanodrop), and RNA integrity was assessed using the Agilent 2100 bioanalyzer. Gene quantification was performed on the NanoString nCounter platform per the manufacturer protocols, using a lab-developed customized 103-gene clinical immuno-oncology panel. Raw NanoString data (RCC files) were processed using the NanoStringQCPro package (version 1.26.0). For quality control, the geometric mean of four housekeeping genes (*DDX50, EIF2B4, MRPS5,* and *SAP130*) was used as a metric of overall RNA quality and quantity, and samples with a value of <100 were excluded. The data from all samples were normalized together using housekeeping genes and positive control genes as recommended in the NanoString data analysis guidelines. Briefly, for each sample, a normalization was performed sequentially using first the positive controls and then the housekeeping genes. For each sample, the geometric mean of the positive controls was calculated (geomeancontrol). The geometric mean of all the geometric means was then calculated (geomeangeomeans), and this was used to calculate a sample-specific normalization factor (geomeangeomeans/geomeancontrol), which was multiplied by the gene counts for each sample. The same steps were then taken using the housekeeping genes instead of the positive controls, to provide the final normalized gene counts. To provide an overall assessment of the immune microenvironment, we used the tumor inflammation signature (TIS), which is also referred to in the literature as “T-cell inflamed gene expression profile.” This is a well-validated 18 gene score that includes probes involved in antigen-presenting cell abundance (*PSMB10, HLA-DQA1,* and *HLA-DRB1*), T cell/NK cell abundance (*HLA-E, NKG7,* and *CD8A*), interferon activity (*CCL5, CXCL9, CD27, CXCR6, IDO1,* and *STAT1*), and T-cell exhaustion (*TIGIT, LAG3, CD274, PDCD1LG2,* and *CD276*). The scores for each sample were calculated using matrix multiplication of the log_2_-transformed gene counts by the corresponding weights for each gene from the reference paper (65, 66). Heat maps were created using ComplexHeatmap (version 2.10.1; ref. 67).

For IHC analysis of the microenvironment, 4-μm thick sections of FFPE surgical specimens were stained using an automated stainer (Dako OMNIS) with the following primary antibodies: PD-L1 (clone28-8, Abcam, 1/500) and CD8 (Clone c8/144B, Dako OMINS). IHC analysis was performed by a board-certified neuropathologist (AL) blinded to patient outcomes. CD8 cells were quantified as the number of positive cells in the top 1 mm^2^ of tissue (5 regions selected for maximum cell density). PD-L1 was quantified visually as >1% or <1% positive staining in tumor or antigen-presenting cells.

### TCR Rearrangement Repertoire Profiling

Genomic DNA was extracted (Methods as above) from serial peripheral blood samples. Library preparation and capTCRseq hybrid capture were performed (Mulder 2018). Following library preparation, the samples were sequenced first on a MiSeq for QC purposes and then 300 ng of each sample, pooled in a ratio of 1:1:1, was processed for a 3-step capture using a target hybrid capture panel. Post-capture QC was performed on a MiSeq, followed by sequencing of up to a depth of ∼2 million reads on the NextSeq. Post-sequencing, the raw data were analyzed using MiXCR version 2.1.12, “iNext,” “immunarch” R packages, and Pugh Lab customized functions to look at TCR rearrangements in the form of unique clonotypes (VDJ rearranged sequences) for T-cell receptors alpha, beta, gamma, and delta. As the total read depth varied across the cohort, affecting the total successfully aligned reads, all raw fastq reads were down-sampled to ∼1 million reads. QC parameters of percent aligned reads, reads used in clonotypes, final clonotype count, and the total number of clonotypes per 1,000 reads were considered.

### Statistical Analyses and Illustrations

Statistical comparisons were performed using the Fisher exact test and the Wilcoxon–Mann–Whitney test, for parametric and nonparametric data, respectively. Survival analyses were performed using the Kaplan–Meier method. To specifically understand the impact of immunotherapy on survival outcomes, the survival time was calculated from the start of salvage treatment initiation, as occasionally there were delays between detection of radiologic progression and initiation of treatment. The initial progression-free survival on treatment (PFS1) was estimated from the time of ICI monotherapy initiation to radiologic progression. The first overall survival (OS1) was estimated from the time of ICI monotherapy initiation to death from any cause or last follow-up for those alive. The second progression-free survival (PFS2) was estimated from the time of initiation of the salvage therapies to clinical or radiographic progression or death. Survival probability (OS2) was defined from the time of initiation of the salvage regimen to death from any cause or the last follow-up for those alive. Log-rank test was used for comparison between groups. All *P* values were two-sided, with a cutoff of 0.05 for significance. Statistical analyses were performed with R.v.3.5. The generated plots were edited for aesthetics using Adobe Illustrator v.23.0.1.

### Data Availability

Data are available from the corresponding authors upon request linked to a relevant research proposal that will need to be reviewed and approved by the IRRDC scientific committee. The data are not publicly available because of information that could compromise the privacy of the research participants.

## Supplementary Material

Table S1 Baseline characteristics of patients with RRD high-grade gliomas treated with immune-checkpoint inhibition (ICI) (n=75)

Table S2 Demographics and details of treatment in patients who continued ICI progression (n=38)

Supplementary Figures S1-S5 Supplementary Figures S1 to S5, with each figure followed by its corresponding legend in the next page
