# Supplementary material for: Combined Immunotherapy Improves Outcome for Replication-Repair-Deficient (RRD) High-Grade Glioma Failing Anti–PD-1 Monotherapy: A Report from the International RRD Consortium
Source: Cancer Discov. 2023 Oct 12;14(2):258–73. doi: 10.1158/2159-8290.CD-23-0559 (PMC10850948; doi:10.1158/2159-8290.CD-23-0559)
Supplement: Table S1 — Baseline characteristics of patients with RRD high-grade gliomas treated with immune-checkpoint inhibition (ICI) (n=75) [file cd-23-0559_table_s1_suppst1.docx]

**Supplement Table S1. Baseline characteristics of patients with RRD high-grade gliomas treated with immune-checkpoint inhibition (ICI) (n=75)**

| **Characteristics** | **Entire cohort (n=75)** | **Not progressed (n=20)** | **Progressed (n=55)** | **p-value** |
| --- | --- | --- | --- | --- |
| **Median age (IQR) (years)** | 12.74 (9.3; 15.7) | 10.18 (6.9; 15.1) | 12.7 (9.3; 15.7) | 0.18 |
| **Sex** |  |  |  |  |
| Male | 36 (48%) | 8 | 28 | 0.44 |
| Female | 39 (52%) | 12 | 27 |  |
| **Germline predisposition** |  |  |  |  |
| Constitutional mismatch repair deficiency | 53 (71%) | 14 | 39 | 1.00 |
| Lynch syndrome | 19 (25%) | 5 | 14 |  |
| Polymerase proofreading associated polyposis | 1 (1.3%) | 0 | 1 |  |
| None/ Somatic | 2 (2.7%) | 1 | 1 |  |
| **WHO grade** |  |  |  |  |
| Grade 3 | 9 (12%) | 2 | 7 | 1.00 |
| Grade 4 | 66 (88%) | 18 | 48 |  |
| **Median TMB (IQR) (mutations/Mb)** | 190 (15.8; 346.8) | 280.5 (214; 395.5) | 190 (15.8; 346.8) | 0.07 |
| **ICI agent used** |  |  |  |  |
| Nivolumab | 60 (80%) | 18 | 42 | 0.32 |
| Pembrolizumab | 14 (20%) | 2 | 12 |  |
| Durvalumab | 1 (1.3%) | 0 | 1 |  |

Abbreviations: IQR: Interquartile range; TMB: Tumor mutation burden; WHO: World Health Organisation, Mb: megabase
