# Supplementary material for: Combined Immunotherapy Improves Outcome for Replication-Repair-Deficient (RRD) High-Grade Glioma Failing Anti–PD-1 Monotherapy: A Report from the International RRD Consortium
Source: Cancer Discov. 2023 Oct 12;14(2):258–73. doi: 10.1158/2159-8290.CD-23-0559 (PMC10850948; doi:10.1158/2159-8290.CD-23-0559)
Supplement: Table S2 — Demographics and details of treatment in patients who continued ICI progression (n=38) [file cd-23-0559_table_s2_suppst2.docx]

**Supplementary Table S2. Demographics and details of treatment in patients who continued ICI progression (n=38)**

| **ID** | **Age (years)** | **Sex** | **Germline** | **Grade** | **TMB (mut/Mb)** | **Initial ICI** | **Prior ICI treatment (months)** | **Pattern of failure** | **Salvage treatment** | **Additional radiation** | **Toxicity > CTCAE 2** | **Immune side effects** | **Progression** | **PFS2 (months)** | **New cancers** | **Alive/Dead** | **Cause of death** | **OS2 (months)** |
| --- | --- | --- | --- | --- | --- | --- | --- | --- | --- | --- | --- | --- | --- | --- | --- | --- | --- | --- |
| 1 | 5.03 | Male | CMMRD | 4 | 496 | Nivolumab | 7.30 | Local | Ipilimumab + Nivolumab | Yes | Yes | Gastritis, pneumonitis | Did not progress | 48.62 | Second glioma | Dead | Second glioma | 48.62 |
| 2 | 18.12 | Male | CMMRD | 3 | 16 | Pembrolizumab | 3.39 | Local | Ipilimumab + Nivolumab | Yes | Yes | Hepatitis | Further progression | 6.97 | *IDH*-WT GBM | Dead | Primary glioma | 33.04 |
| 3 | 34.29 | Male | PPAP | 4 | 320 | Pembrolizumab | 35.87 | Both | Ipilimumab + Pembrolizumab | Yes | No | None reported | Further progression | 12.99 | Embryonal tumor | Alive | – | 22.95 |
| 4 | 11.41 | Female | CMMRD | 4 | 834 | Nivolumab | 24.99 | Distant | Ipilimumab + Nivolumab | Yes | No | None reported | Did not progress | 16.06 | No | Alive | – | 16.08 |
| 5 | 15.33 | Male | CMMRD | 4 | 191 | Nivolumab | 18.12 | Local | Ipilimumab + Nivolumab | No | Yes | Colitis, hepatitis, nephritis | Further progression | 2.04 | No | Dead | Primary glioma | 14.07 |
| 6 | 10.08 | Female | CMMRD | 4 | 15.5 | Nivolumab | 0.30 | Local | Ipilimumab + Nivolumab | No | No | Hypothyroidism | Further progression | 12.00 | No | Dead | Primary glioma | 13.87 |
| 7 | 13.11 | Female | Lynch | 3 | 15.4 | Nivolumab | 8.32 | Local | Ipilimumab + Nivolumab | Yes | Yes | None | Further progression | 6.00 | No | Dead | Primary glioma | 12.26 |
| 8 | 17.51 | Female | CMMRD | 4 | 518 | Nivolumab | 25.58 | Distant | Ipilimumab + Nivolumab | No | No | Fatigue | Further progression | 2.66 | No | Dead | Primary glioma | 12.10 |
| 9 | 9.03 | Male | CMMRD | 4 | 385.9 | Nivolumab | 12.00 | Distant | Ipilimumab + Nivolumab | No | No | None reported | Further progression | 9.00 | No | Dead | Primary glioma | 11.41 |
| 10 | 17.28 | Female | CMMRD | 3 | 7.4 | Nivolumab | 19.23 | Distant | Ipilimumab + Nivolumab | No | Yes | Hepatitis | Further progression | 5.00 | No | Dead | Primary glioma | 10.39 |
| 11 | 13.95 | Female | CMMRD | 4 | 295 | Nivolumab | 47.01 | Local | Ipilimumab + Nivolumab | Yes | Yes | Hepatitis | Did not progress | 6.94 | No | Alive | – | 6.94 |
| 12 | 16.78 | Male | CMMRD | 4 | 24.83 | Pembrolizumab | 21.07 | Local | Ipilimumab + Pembrolizumab | Yes | Yes | Hepatitis, colitis | Did not progress | 5.98 | Lymphoma | Dead | Lymphoma | 5.98 |
| 13 | 17.82 | Female | Lynch | 4 | 21.53 | Nivolumab | 16.50 | Local | Ipilimumab + Nivolumab | No | Yes | Colitis | Further progression | 3.25 | No | Dead | Primary glioma | 4.93 |
| 14 | 15.84 | Female | Lynch | 4 | 73 | Nivolumab | 5.26 | Local | Ipilimumab + Nivolumab | Yes | No | None reported | Did not progress | 4.57 | No | Alive | – | 4.57 |
| 15 | 10.81 | Female | Lynch | 4 | 11.2 | Pembrolizumab | 6.31 | Local | Ipilimumab + Pembrolizumab | No | No | None reported | Further progression | 0.46 | No | Dead | Primary glioma | 3.02 |
| 16 | 11.44 | Female | CMMRD | 3 | 189 | Nivolumab | 1.64 | Local | Ipilimumab + Nivolumab | No | No | None reported | Further progression | 0.46 | No | Dead | Primary glioma | 2.96 |
| 17* | 10.66 | Male | CMMRD | 4 | 432.8 | Pembrolizumab | 19.03 | Distant | Ipilimumab + Nivolumab | Yes | Yes | Thrombosis | Did not progress | 2.93 | Lymphoma | Alive | – | 2.93 |
| 18 | 9.80 | Female | CMMRD | 4 | – | Nivolumab | 7.13 | Local | Ipilimumab + Nivolumab | No | Yes | Hepatitis | Did not progress | 2.86 | No | Alive | Primary glioma | 2.86 |
| 19 | 17.02 | Male | Lynch | 4 | 25.92 | Nivolumab | 10.88 | Local | Ipilimumab + Nivolumab | No | Yes | Colitis | Further progression | 1.55 | No | Alive |  | 2.37 |
| 20 | 18.06 | Male | Lynch | 4 | 9.5 | Nivolumab | 10.03 | Local | Ipilimumab + Nivolumab | No | No | None reported | Did not progress | 1.94 | No | Alive | – | 1.94 |
| 21 | 10.54 | Female | CMMRD | 4 | 254 | Nivolumab | 9.13 | Local | Ipilimumab + Nivolumab | No | No | None reported | Did not progress | 1.81 | No | Alive | – | 1.81 |
| 22 | 7.67 | Female | CMMRD | 4 | 541 | Nivolumab | 6.61 | Local | Ipilimumab + Nivolumab | No | No | None reported | Further progression | 0.46 | No | Dead | Primary glioma | 1.61 |
| 23 | 9.72 | Male | CMMRD | 4 | 387 | Pembrolizumab | 25.01 | Local | Ipilimumab + Nivolumab | No | No | None reported | Did not progress | 0.95 | No | Alive | – | 0.95 |
| 24* | 7.57 | Male | CMMRD | 4 | 208.3 | Nivolumab | 8.12 | Local | Ipilimumab + Nivolumab | No | No | None reported | Did not progress | 0.89 | No | Alive | – | 0.89 |
| 25* | 12.68 | Male | CMMRD | 4 | 261 | Nivolumab | 5.52 | Local | Trametinib + Nivolumab | Yes | Yes | Weight gain, paronychia, mucositis | Further progression | 13.81 | No | Dead | Primary glioma | 15.52 |
| 26 | 11.48 | Male | CMMRD | 4 | 183 | Nivolumab | 4.64 | Local | Trametinib + Nivolumab | Yes | Yes | Paronychia | Further progression | 6.21 | No | Dead | Primary glioma | 10.26 |
| 27 | 18.23 | Male | CMMRD | 4 | 181.66 | Nivolumab | 14.50 | Both | Trametinib + Nivolumab | No | Yes | Acne, pustular lesions, paronychia, ear bleeds | Further progression | 2.56 | No | Dead | Primary glioma | 8.22 |
| 28 | 13.40 | Female | None/ Somatic | 4 | 10 | Nivolumab | 7.79 | Local | Trametinib + Nivolumab | Yes | No | None reported | Did not progress | 3.91 | No | Alive | – | 3.91 |
| 29 | 14.31 | Male | CMMRD | 3 | 377 | Nivolumab | 13.51 | Local | Trametinib + Nivolumab | No | No | None reported | Further progression | 0.99 | No | Dead | Primary glioma | 2.01 |
| 30 | 15.31 | Male | CMMRD | 4 | 318 | Pembrolizumab | 8.12 | Distant | Pembrolizumab | Yes | Yes | Pneumonitis, pancreatitis | Further progression | 5.39 | No | Dead | Primary glioma | 12.20 |
| 31 | 7.71 | Female | CMMRD | 4 | 452 | Nivolumab | 33.08 | Distant | Nivolumab | Yes | No | None reported | Further progression | 9.96 | No | Dead | Primary glioma | 11.64 |
| 32 | 47.54 | Male | Lynch | 3 | 13.2 | Pembrolizumab | 50.40 | Distant | Pembrolizumab | Yes | No | None reported | Did not progress | 2.93 | No | Alive | – | 2.93 |
| 33 | 15.84 | Female | Lynch | 4 | 17 | Pembrolizumab | 2.90 | Local | Pembrolizumab | Yes | No | None reported | Did not progress | 6.94 | No | Alive | – | 6.94 |
| 34 | 10.14 | Female | CMMRD | 4 | 559 | Nivolumab | 3.00 | Local | Nivolumab | No | No | None reported | Did not progress | 14.96 | No | Alive | – | 14.96 |
| 35 | 3.31 | Male | CMMRD | 4 | 275 | Nivolumab | 2.50 | Local | Nivolumab | No | No | None reported | Did not progress | 11.34 | No | Alive | – | 11.34 |
| 36 | 28.03 | Female | CMMRD | 4 | – | Nivolumab | 2.80 | Local | Pembrolizumab | No | No | None reported | Further progression | 3.68 | No | Dead | Primary glioma | 5.50 |
| 37 | 10.88 | Male | CMMRD | 4 | 60 | Nivolumab | 1.38 | Both | Nivolumab | No | No | None reported | Further progression | 1.41 | No | Alive | Primary glioma | 1.55 |
| 38 | 13.92 | Male | CMMRD | 4 | 800 | Nivolumab | 16.47 | Local | Nivolumab | No | No | None reported | Did not progress | 1.35 | No | Alive | – | 1.35 |

Abbreviations: CMMRD: Constitutional Mismatch Repair Deficiency Syndrome; PPAP: Polymerase-proofreading associated polyposis; TMB: Tumor mutation burden (mutations/megabase); ICI: Immune checkpoint inhibition; CTCAE: Common Terminology Criteria for Adverse Events. (*: Patients who initially got ICI treatment as primary adjuvant therapy before progression of their glioma).
