# Supplementary material for: Combined Immunotherapy Improves Outcome for Replication-Repair-Deficient (RRD) High-Grade Glioma Failing Anti–PD-1 Monotherapy: A Report from the International RRD Consortium
Source: Cancer Discov. 2023 Oct 12;14(2):258–73. doi: 10.1158/2159-8290.CD-23-0559 (PMC10850948; doi:10.1158/2159-8290.CD-23-0559)
Supplement: Supplementary Figures S1-S5 — Supplementary Figures S1 to S5, with each figure followed by its corresponding legend in the next page [file cd-23-0559_supplementary_figures_s1-s5_suppsf1.pdf]

Fig.S1

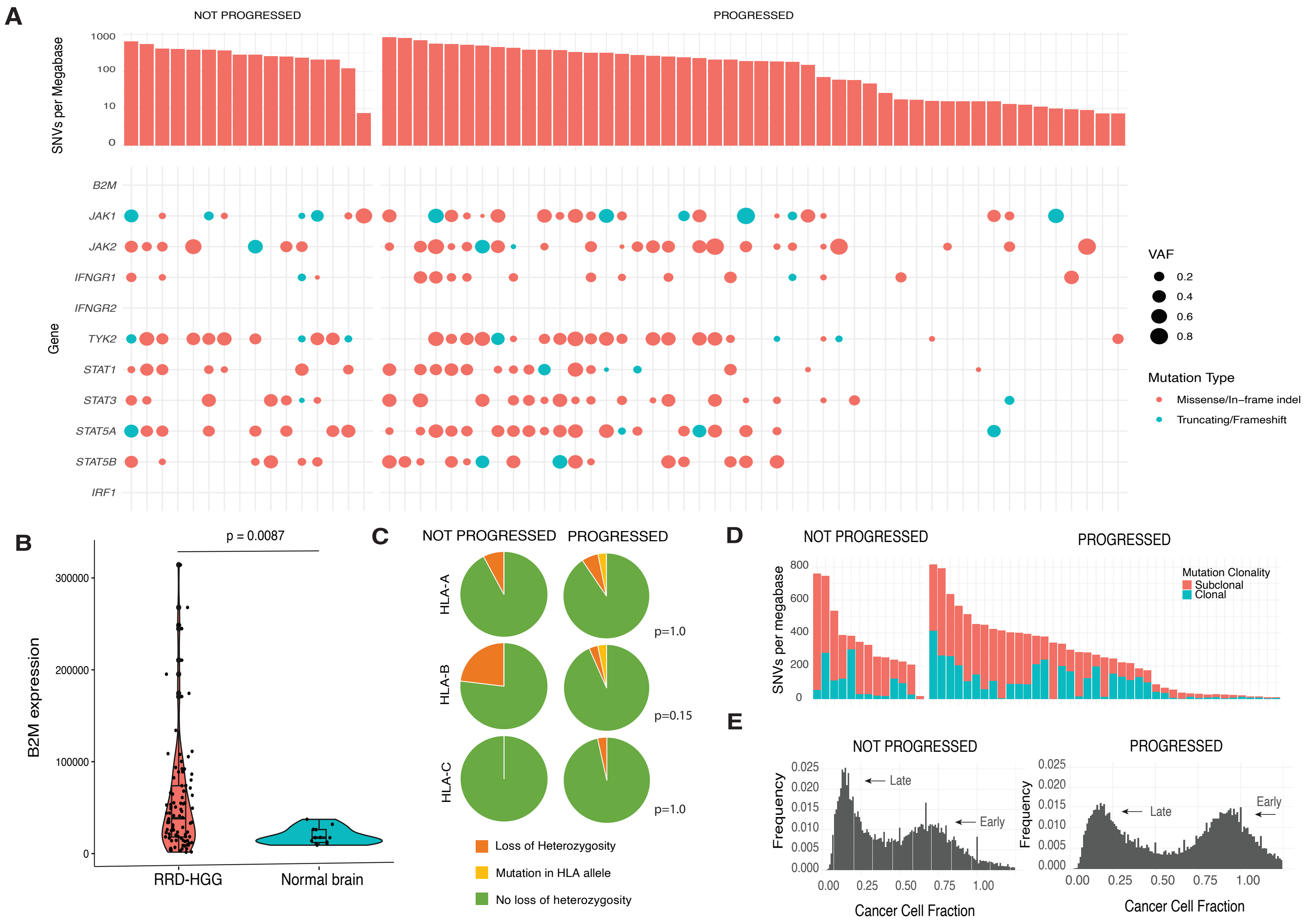
A
NOT PROGRESSED
PROGRESSED
SNVs per Megabase
1000
100
10
0
Gene
B2M
JAK1
JAK2
IFNGR1
IFNGR2
TYK2
STAT1
STAT3
STAT5A
STAT5B
IRF1
VAF
0.2
0.4
0.6
0.8
Mutation Type
Missense/In–frame indel
Truncating/Frameshift
B
p = 0.0087
B2M expression
300000
200000
100000
0
RRD-HGG
Normal brain
C
NOT PROGRESSED
PROGRESSED
HLA-A
HLA-B
HLA-C
p=1.0
p=0.15
p=1.0
Loss of Heterozygosity
Mutation in HLA allele
No loss of heterozygosity
D
NOT PROGRESSED
PROGRESSED
SNVs per megabase
800
600
400
200
0
Mutation Clonality
Subclonal
Clonal
E
NOT PROGRESSED
PROGRESSED
Frequency
0.025
0.020
0.015
0.010
0.005
0.000
Late
Early
0.00
0.25
0.50
0.75
1.00
Cancer Cell Fraction

Supplemental Fig.S1. (A) Absence of enrichment of mutations in genes related to antigen presentation and interferon signalling in patients who progressed on anti-PD(L)1 and those who did not progress. Specifically, there were no mutations detected at all in the *B2M* gene, and no previously reported pathogenic or likely pathogenic mutations were detected in *JAK1/ JAK2* in these hypermutant RRD-HGG on whole exome analysis (variant allele frequency cut-off: 0.05). **(B)** B2M expression is uniformly elevated in all RRD-HGG on gene expression using the Nanostring-3D platform as compared to control, non-malignant brain tissue from non-CMMRD/Lynch patients. **(C)** Absence of difference in loss of heterozygosity (LOH) for HLA-A, B and C between progressed and non-progressed patients ($p>0.1$) (Methods). A single patient harboured a somatic mutation in both HLA-A (HLA-A*02:03/HLA-A*69:01 to HLA-A*02:01/HLA-A*69:01) and HLA-B (HLA-B*55:01/HLA-B*52:01 to HLA-B*59:01/HLA-B*78:01). **(D)** Clonal vs sub-clonal mutations showed no differential pattern between those who progressed versus those who did not (variant allele frequency cut-off of 0.85 was used to classify as 'clonal'). **(E)** A relatively more prominent peak of late burst of mutations in those who did not progress on anti-PD(L)1 therapy. This late-burst (arrows) is associated with secondary somatic *POLE/POLD1* mutations in RRD-cancers[11]. In contrast, enrichment for an early-burst of mutations (related to the primary MMR-deficiency) and lower peak of the second burst was seen in patients who did not progress on anti-PD(L)1 treatment. *POLE/POLD1* mutations are associated with favourable responses to ICI treatment[16,29].

Fig.S2

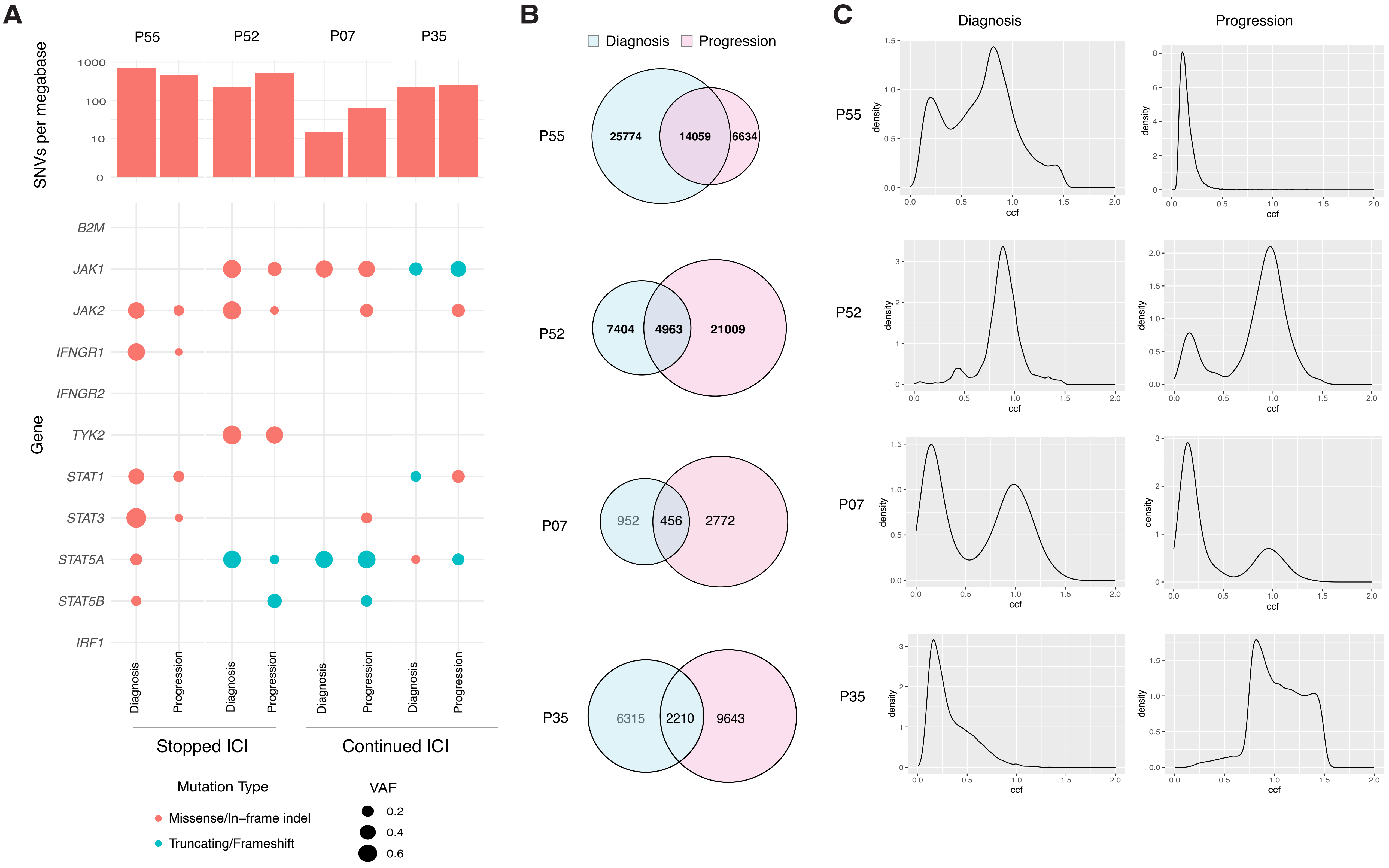
A
B
C
P55
P52
P07
P35
SNVs per megabase
1000
100
10
0
Gene
B2M
JAK1
JAK2
IFNGR1
IFNGR2
TYK2
STAT1
STAT3
STAT5A
STAT5B
IRF1
Diagnosis
Progression
Stopped ICI
Continued ICI
Mutation Type
Missense/In−frame indel
Truncating/Frameshift
VAF
0.2
0.4
0.6
25774
14059
6634
7404
4963
21009
952
456
2772
6315
2210
9643
density
ccf

Supplemental Fig.S2. **(A)** Absence of enrichment of mutations in genes related to antigen presentation and interferon signalling in patients who had a second specimen after progression following anti-PD1 monotherapy*. **(B)** Venn-diagram showing mutational overlap at the two time-points in all four patients showed definite evolution in mutational spectrum. **(C)** Histograms depicting the cancer cell fraction (CCF) shows variable patterns of clonal evolution at the time of progression on anti-PD1 therapy suggesting stochastic mutation accumulation. [*Two patients stopped ICI and had died (post-mortem samples; P55, P52). Two patients (P07, P35) had continued ICI after a biopsy and showed delayed responses (Fig.S5, Fig.6)].

Fig.S3

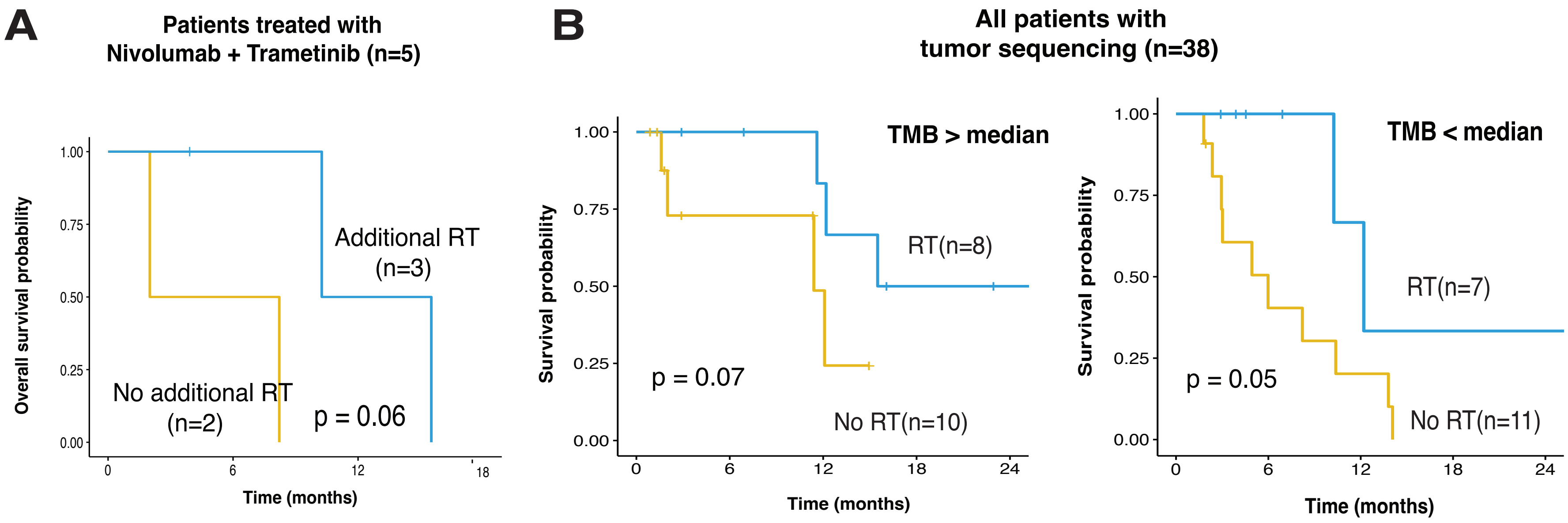
A
Patients treated with Nivolumab + Trametinib (n=5)
Overall survival probability
1.00
0.75
0.50
0.25
0.00
Additional RT (n=3)
No additional RT (n=2)
p = 0.06
0
6
12
18
Time (months)
B
All patients with tumor sequencing (n=38)
Survival probability
TMB > median
RT(n=8)
No RT(n=10)
p = 0.07
0
6
12
18
24
Time (months)
Survival probability
TMB < median
RT(n=7)
No RT(n=11)
p = 0.05
0
6
12
18
24
Time (months)

Supplement Fig.S3: (A) Impact of re-irradiation in a pilot cohort of patients who also received MEK-inhibitor with anti-PD1 at progression. **(B)** Impact of re-irradiation in RRD-HGG stratified by tumor mutation burden (TMB) exceeding the mean (RT: radiation).

Fig.S4

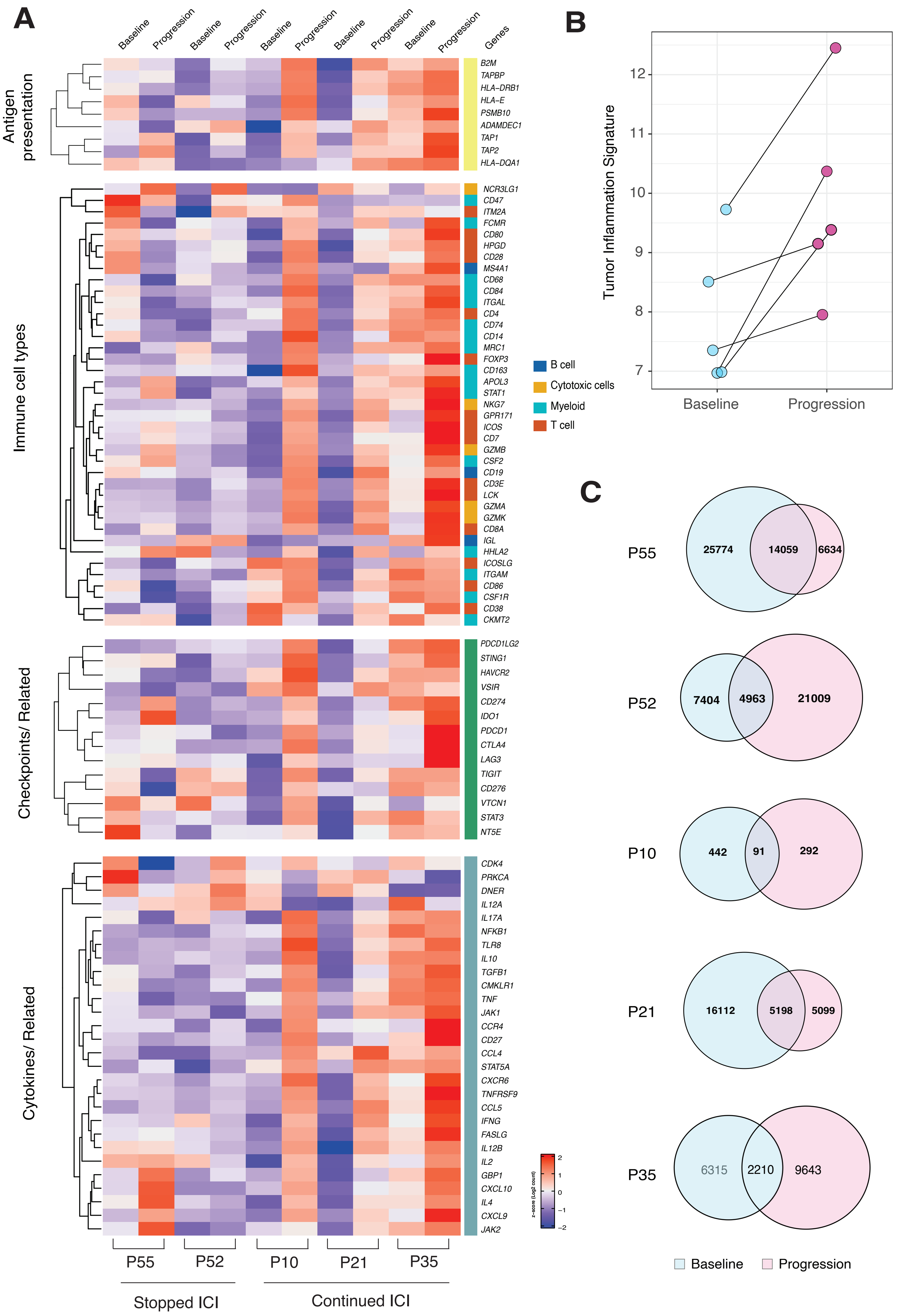
A
Baseline
Progression
Genes
Antigen presentation
B2M
TAPBP
HLA-DRB1
HLA-E
PSMB10
ADAMDEC1
TAP1
TAP2
HLA-DQA1
Immune cell types
NCR3LG1
CD47
ITM2A
FCMR
CD80
HPGD
CD28
MS4A1
CD68
CD84
ITGAL
CD4
CD74
CD14
MRC1
FOXP3
CD163
APOL3
STAT1
NKG7
GPR171
ICOS
CD7
GZMB
CSF2
CD19
CD3E
LCK
GZMA
GZMK
CD8A
IGL
HHLA2
ICOSLG
ITGAM
CD86
CSF1R
CD38
CKMT2
B cell
Cytotoxic cells
Myeloid
T cell
Checkpoints/ Related
PDCD1LG2
STING1
HAVCR2
VSIR
CD274
IDO1
PDCD1
CTLA4
LAG3
TIGIT
CD276
VTCN1
STAT3
NT5E
Cytokines/ Related
CDK4
PRKCA
DNER
IL12A
IL17A
NFKB1
TLR8
IL10
TGFB1
CMKLR1
TNF
JAK1
CCR4
CD27
CCL4
STAT5A
CXCR6
TNFRSF9
CCL5
IFNG
FASLG
IL12B
IL2
GBP1
CXCL10
IL4
CXCL9
JAK2
z-score (Log2 count)
2
1
0
−1
−2
P55
P52
P10
P21
P35
Stopped ICI
Continued ICI
B
Tumor Inflammation Signature
12
11
10
9
8
7
Baseline
Progression
C
P55
25774
14059
6634
P52
7404
4963
21009
P10
442
91
292
P21
16112
5198
5099
P35
6315
2210
9643
Baseline
Progression

Supplementary Fig.S4. (A) Gene expression using the Nanostring-3D immune panel in five patients with samples at diagnosis and relapse*. The expression profile suggests overall increase for multiple pro-inflammatory markers including those related to antigen presentation, immune cell infiltration, compensatory checkpoint upregulation and cytokine activation. **(B)** The 18-gene tumor inflammation signature (TIS) score (Methods)[66,67] showed progressive increase over time in all five patients. Higher TIS is associated with improved immune checkpoint inhibitor response. **(C)** Mutational spectrum compared using Venn diagram between baseline and progression for the five patients shows evolution over time. [*Two patients (P55, P52) had stopped ICI and the second sample was post-mortem. Time-point for progression for P21 is at relapse but prior to anti-PD1 initiation and for all others, the second samples are after anti-PD1 treatment. Three patients (P10, P21, P35) continued ICI. P10 and P21 had stable disease and P35 had objective response, with both P21 and P35 having prolonged ongoing survival].

Fig.S5

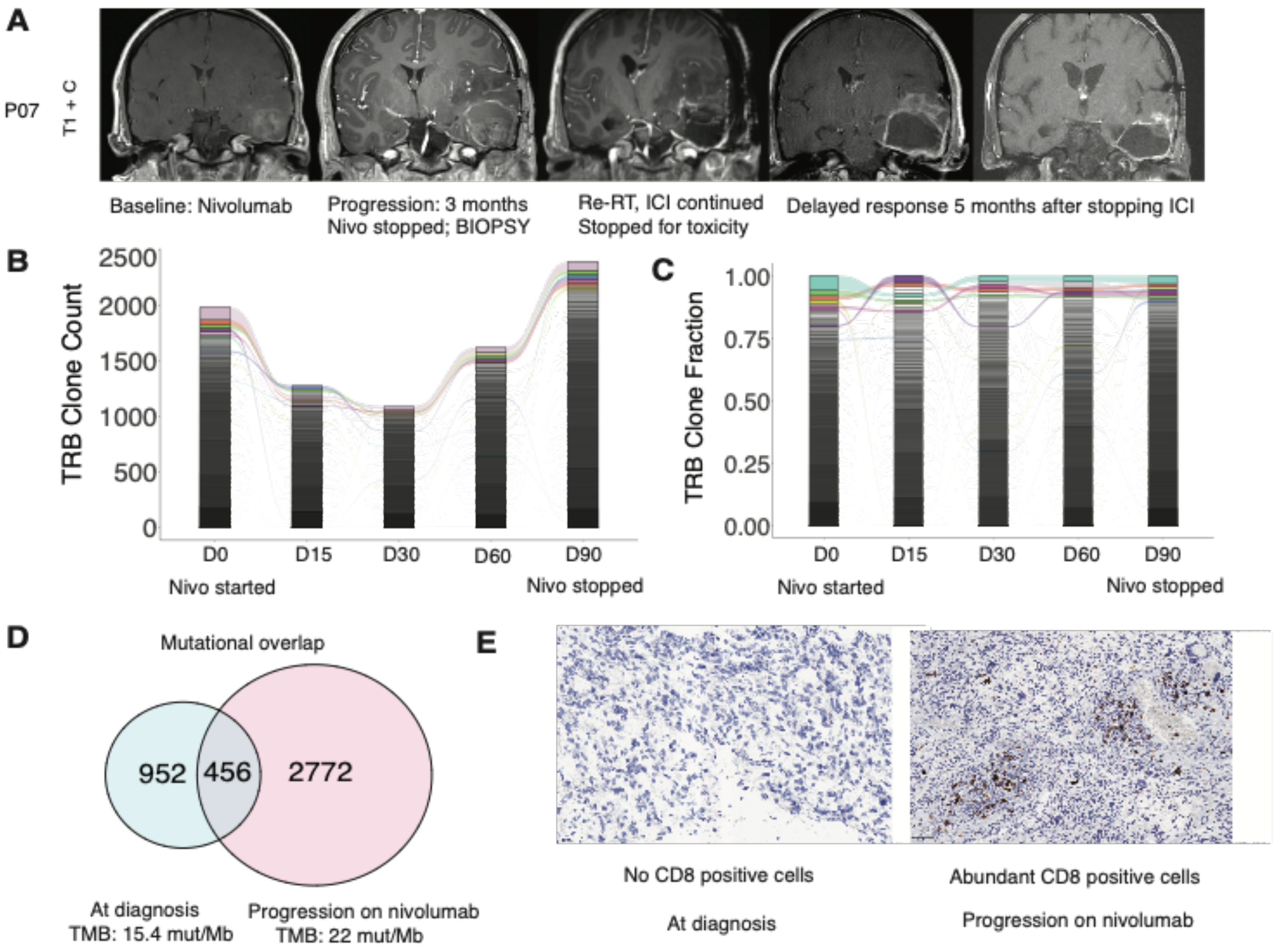
A
P07
T1 + C
Baseline: Nivolumab
Progression: 3 months
Nivo stopped; BIOPSY
Re-RT, ICI continued
Stopped for toxicity
Delayed response 5 months after stopping ICI
B
TRB Clone Count
2500
2000
1500
1000
500
0
D0
D15
D30
D60
D90
Nivo started
Nivo stopped
C
TRB Clone Fraction
1.00
0.75
0.50
0.25
0.00
D0
D15
D30
D60
D90
Nivo started
Nivo stopped
D
Mutational overlap
952
456
2772
At diagnosis
TMB: 15.4 mut/Mb
Progression on nivolumab
TMB: 22 mut/Mb
E
No CD8 positive cells
At diagnosis
Abundant CD8 positive cells
Progression on nivolumab

Supplement Fig.S5. (A) Clinical course and serial radiology for patient P7. **(B)** At the time of stopping nivolumab treatment, the T-cell receptor (TCR) clonotype analysis using Cap-TCR-sequencing demonstrated an increasing trajectory of clone count, with **(C)** enrichment of specific clones. **(D)** A biopsy performed immediately after showed increase in TMB and emergence of new mutations, with overlap between primary and second sample shown as a Venn diagram. **(E)** This was associated with increase in CD8+ T-cell infiltration in the microenvironment and delayed response on radiology.
